# Treatment with captopril abrogates the altered expression of alpha1 macroglobulin and alpha1 antiproteinase in sera of spontaneously hypertensive rats

**DOI:** 10.1186/1477-5956-10-17

**Published:** 2012-03-15

**Authors:** Norhaniza Aminudin, Nur-Atiqah H Abdullah, Hasni Misbah, Saiful A Karsani, Ruby Husain, See Z Hoe, Onn H Hashim

**Affiliations:** 1 Institute of Biological Sciences, Faculty of Science, University of Malaya, 50603 Kuala Lumpur, Malaysia; 2 University of Malaya Centre for Proteomics Research, University of Malaya, Kuala Lumpur, Malaysia; 3 Department of Molecular Medicine, Faculty of Medicine, University of Malaya, Kuala Lumpur, Malaysia; 4 Department of Physiology, Faculty of Medicine, University of Malaya, Kuala Lumpur, Malaysia

**Keywords:** hypertension, biomarker, spontaneously hypertensive rats, serum proteins, proteomics

## Abstract

**Background:**

Proteins that are associated with hypertension may be identified by comparing the 2-dimensional gel electrophoresis (2-DE) profiles of the sera of spontaneously hypertensive rats (SHR) with those generated from normotensive Spraque-Dawley rats (SDR).

**Results:**

Five proteins of high abundance were found to be significantly altered when the 2-DE serum profiles of the SHR were compared to those that were similarly generated from the SDR. Analysis by mass spectrometry and database search identified the proteins as retinol binding protein 4, complement C3, albumin (19.9 kDa fragment), alpha1 macroglobulin and alpha1 antiproteinase, which are all known to be associated with hypertension. The altered expression of the two latter proteins was found to be abrogated when similar analysis was performed on sera of the SHR that were treated with captopril.

**Conclusion:**

Our data suggests that serum alpha1 macroglobulin and alpha1 antiproteinase are potentially useful complementary biomolecular indicators for monitoring of hypertension.

## Background

Spontaneously hypertensive rats (SHR) have been widely used as an animal model to investigate primary hypertension and its relationship to cardiovascular diseases. The SHR strain was generated in the 1960s by Okamoto et al. by selective breeding of the Wistar-Kyoto rats with high blood pressure [1]. The blood pressure of SHR usually rises at around 5-6 weeks of age and the systolic pressure of an adult SHR may reach a value of between 180 and 200 mmHg. The SHR usually develops characteristics of cardiovascular diseases like hypertrophy of the heart and blood vessels, which start at around 40 weeks of age [2].

Hypertension has been known to cause the altered levels of serum or plasma proteins. A considerable number of proteins have been previously reported to be altered in levels in the sera of both animals and human subjects [3-6]. While some of the proteins were thought to be involved as anti-inflammatory and protective response [4,7], others were related to endothelial vascular repair [5], arterial smooth muscle cell growth [6] and some may instead be the contributing factors of hypertension.

In the present study, we have investigated the simultaneous expression of the high abundant serum proteins in the normotensive Spraque-Dawley rats (SDR) and compared it with those expressed in the sera of SHR as well as the SHR that were treated with captopril.

## Results

### Monitoring of rat blood pressure

Table 1 demonstrates the blood pressure of control rats and those treated with 60 mg/kg body weight/day of captopril. The blood pressure of the control SDR rat group showed stable normal systolic pressure. The hypertensive group of rats (SHR) also showed an unchanged high level of systolic blood pressure. However, a significant reduction of the blood pressure was observed in the captopril-treated SHR.

**Table 1:** Mean systolic blood pressure of control and captopril-treated rats

Group	Systolic blood pressure (mmHg)	
	**Day 1**	**Day 10**

**SDR**	102 ± 2.0	103 ± 3.4

**SHR**	197 ± 6.8	196 ± 6.8

**C-SHR**	191 ± 4.0	151 ± 8.9

SDR - Sprague Dawley rats, SHR - spontaneously hypertensive rats, C-SHR - captopriltreated SHR. Data are expressed as mean ± S.E.M. Values of *p *< 0.05 were considered significant.

### Profiling of rat serum samples

**Figure 1:**
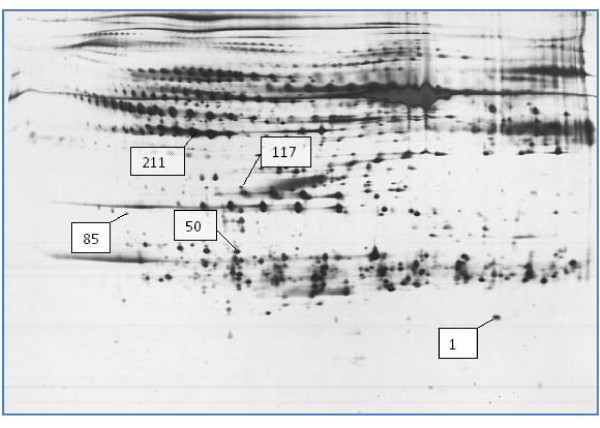
**Image of a representative silver-stained 2-DE gel showing the high abundant proteins detected in an SHR serum**. Spots 1, 50, 85, 117 and 211 were significantly altered in levels when image analysis comparing the expression of 2-DE protein spots of SDR serum samples with those of the SHR. Acid side of the gel is to the left and relative molecular mass declines from the top.

Figure 1 demonstrates a typical representative 2-DE protein profile that was generated from a serum sample obtained from control non-treated SHR. Serum proteins were separated on the basis of their different p*I*s and molecular weights when subjected to 2-DE. Hundreds of highly-resolved protein spots were detected when the 2-DE gels were silver-stained. Due to the broad dynamic range of proteins that are present in the serum, only the highly abundant proteins were detected by silver staining. Similar profiles were generated when serum samples of the SDR and captopril-treated SHR were subjected to 2-DE.

### Image analysis of rat serum proteins

**Figure 2:**
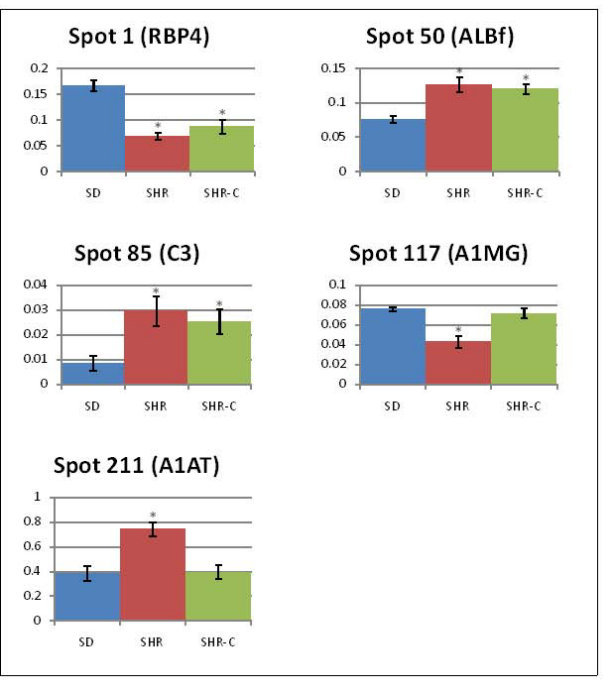
**Relative expression of protein spots of interest in sera of SDR, SHR and captopril-treated SHR**. The percentage of volume contribution was determined using the Image Master 2D Platinum Software 7.0. Relative volumes of spots 1, 50, 85, 117 and 211 were significantly different when comparison was made between SHR and SDR. However, only spots 1, 50 and 85 were significantly different when the 2-DE images of captopriltreated SHR were compared to those obtained from SDR. Y axis of histogram indicates percentage of volume contribution. Asterisks denote significantly different values relative to the SDR (*p*< 0.00233).

**Figure 3:**
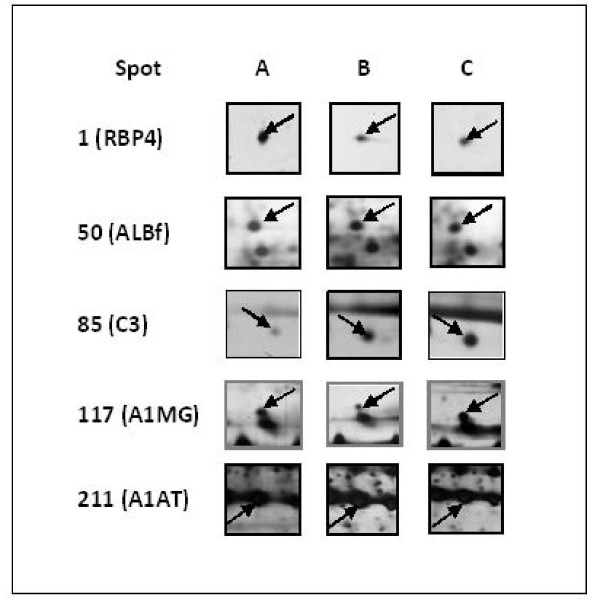
**Representative cropped images of selective protein spots of interest in rat serum**. Panels **A**, **B **and **C **refer to the protein spots detected in the sera of SDR, SHR and Captopril treated SHR (C-SHR), respectively.

When image analysis was performed on the silver-stained 2-DE gels generated using serum samples obtained from the SDR and compared to similar profiles generated from the SHR, the expression of 13 protein spots was initially found to be significantly altered (*p *< 0.05). However, only five of the protein spots, i.e. 1, 50, 85, 117, and 211, were truly altered when the *p*-values were corrected for false significant results using the method of Benjamini and Hochberg [8] (Figure 2). Using the serum proteins expressed in the sera of normotensive SDR as a standard reference, the levels of two proteins were found to be significantly lowered (spots 1 and 117) and three proteins (spots 50, 85 and 211) were apparently enhanced in the sera of the SHR. However, only spots 1, 50 and 85 maintained to be significantly altered in levels when the serum 2-DE profiles of SHR that were treated with captopril were compared those of the normotensive SDR (Figure 2). The expression of spots 117 and 211 was apparently no longer significantly different and appeared to be normalised. Cropped images of the five serum protein spots in 2-DE gels of control and experimental rats are shown in Figure 3.

### Identification of differentially-expressed rat serum proteins

The five differentially expressed protein spots were confidently identified by mass spectrometry using the 4800 Plus MALDI TOF/TOF analyzer and MASCOT database search (Table 2). They were retinol binding protein 4 (RBP4; spot 1), complement C3 (C3; spot 85), albumin (ALB; spot 50), alpha1 macroglobulin (A1MG; spot 117) and alpha 1 antiproteinase (A1AP; spot 211). The albumin spot was apparently resolved in the region of around 19.9 kDa in the 2-DE serum protein profiles, suggesting that it was a fragment of the protein (ALBf).

**Table 2:** Mass spectrometric identification of differentially expressed rat serum proteins

Spot ID	Matched protein identity	Accession number (Swiss-Prot)	Theoretical mass (Da)	Theoretical p*I*	MASCOT score	No. of peptides matched
1	RBP4	P04916	23 205	5.69	596	8

50	ALB	P02770	68 686	6.09	82	10

85	C3	P01026	186 342	6.12	200	5

117	A1MG	P01023	167 019	6.46	547	8

211	A1AT	P17475	46 107	5.70	609	9

Spot ID are as in Figure 1. RBP4, ALB, C3, A1MG and A1AT refer to retinol binding protein 4, albumin, complement C3, alpha1 macroglobulin and alpha1 antiproteinase, respectively. Accession numbers are from the MASCOT database (http://www.matrixscience.com).

## Discussion

In the present study, we firstly demonstrated a significant hypotensive effect of captopril following a 10-day treatment. This acute duration treatment showed a 20% hypotensive response, whereby the baseline blood pressure of 191 mmHg was reduced to 151 mmHg (Table 1). When the sera of SHR were subjected to 2-DE and compared to similar profiles generated from those of SDR, the levels of five protein spots were found to be significantly altered. The spots were subsequently identified as those of the RBP4, C3, a 19.9 kDa fragment of ALB, A1MG and A1AT (Table 2). All proteins have been previously identified to have some association with hypertension. The levels the proteins were earlier reported to be altered in the SHR as well as patients with hypertension, although the experiments were carried out on individual proteins. In this study, the different altered levels of the five serum proteins were detected simultaneously using the gel-based proteomics approach. Interestingly, the altered expression of A1MG and A1AT appeared to be abrogated in the SHR that were treated with captopril as their serum levels were no longer significantly different from those of the SDR.

RBP4 is responsible of delivering retinol to tissues [9]. Many studies have associated the serum/plasma protein with insulin resistance and diabetic complications [10,11]. RBP4 was reported to be elevated in patients with pregnancy-induced hypertension, possibly as a result of perturbed maternal glucose metabolism [12], and in women with hypertension caused by insulin resistance [13]. In contrast to these reports, our results demonstrated a significant reduction of RBP4 in both the non-treated and captopril-treated SHR groups. Treatment of the SHR with captopril did not appear to induce a significant change in the reduced serum levels of RBP4.

C3 is a complement component involved in both the humoral and innate immunity. The levels of C3 were found to be altered in the sera of patients with idiopathic pulmonary arterial hypertension [14], although the nature of this association is unclear. Studies performed by Lin et al. correlated the differential expression of C3 with the enhanced growth of arterial smooth muscle cells from SHR, prior to the development of hypertension [6]. C3 apparently mediates the arterial smooth muscle cell growth in the rats. The data of our study further confirms the altered levels of C3 in SHR. Like RBP4, however, the levels of C3 in the sera of SHR that were treated with captopril remains significantly different from that expressed in the SDR.

Serum ALB levels have been previously associated with hypertension. In an epidemiological investigation, increasing albumin concentration in the serum within the physiological range was found to correlate with the increase in systolic and diastolic blood pressure in men and women in all age groups [15]. However, the albumin spot that was significantly enhanced in both non-treated and captopril-treated SHR compared to SDR in our study appeared to be a 19.9 kDa fragment of the serum protein. These fragments may be a result of the proteolysis of serum albumin, although currently no information is available on the association of serum albumin with hypertension in rats.

A1MG, which is identical to the α2-macroglobulin that is present on the vascular endothelial cells in humans, binds to a group of serine proteases called tonins in rat tissues [16]. Tonin acts on angiotensin I (Ang I), as well as angiotensinogen (AG) and other peptides presenting the N-terminal sequence of AG to form the vasoconstrictor peptide, angiotensin II (Ang II). The tonin-α_1_-macroglobulin complex can generate Ang II from Ang I despite complete inhibition of the Ang I converting enzyme [17]. Studies have demonstrated the capability of tonin to release bradykinin directly, thus suggesting that the protease is involved in the kinintensin system that generates both the pressor (AngII) and depressor (bradykinin) [18]. When taken together, these findings suggest that A1MG may play a role in the regulation of blood pressure. In the present study, the low levels of A1MG detected in the SHR compared to SDR provide some explanation for the increase in blood pressure in the SHR. The lowered levels were apparently normalised when the SHR were treated with captopril, which indicates an inverse correlation of the serum A1MG levels with hypertension.

A1AT is a serine protease inhibitor (serpin) that functions as an antitrypsin as well as an antithrombin [19]. Its primary target includes elastase, plasmin and thrombin. A1AT protects the connective tissues (elastin) from inflammatory enzymes such as elastase in the lungs and pulmonary system, as well as helps to prevent blood coagulation. Levels of the serum acute-phase protein were shown to correlate positively with blood pressure in humans [4].

Similarly, our results indicated that the levels of A1AT were increased in the sera of SHR compared to SDR, suggesting an acute-phase response to the increase of blood pressure in the SHR. Treatment of the SHR with captopril appeared to demonstrate an apparent abrogation of the altered levels of A1AT in sera of the rats, which further suggests a direct correlation of A1AT levels with hypertension.

## Conclusion

Taken together, our data appear to indicate that hypertension causes the different altered expression of RBP4, C3, ALBf, A1MG and A1AT in the rat serum and that the altered levels of the two latter proteins were apparently normalized when the rats were treated with captopril. Unlike the previous results of others that showed similar altered levels of proteins individually, our proteomics analysis was able to demonstrate the altered levels of the proteins simultaneously in the rat serum samples. Together, these proteins have the potential to be used as indicators for monitoring of hypertension and its adverse consequences.

## Materials and methods

### Rat

SHR and SDR aged between 8-10 weeks (weighing 250-320 g) were used throughout the study. In this study, the SDR was used as control normotensive rats as they are less prone to hypertension compared to the WKY. All rats were singly caged and housed under environmentally controlled conditions with 12 hours of light and dark cycles and free access to pellet and water *ad libidum *at the Animal House, Faculty of Medicine, University of Malaya. All treatments were performed according to the standard recommended procedure described in the Helsinki declaration.

### Treatment with captopril

Captopril was solubilised in distilled water (60 mg/kg body weight) and provided to a group of SHR (n = 6) daily for 10 consecutive days in the form of drinking solution (30 ml per rat/day). Untreated SHR (n = 6) and SDR (n = 6) received tap water for drinking. Captopril treated rats will only received normal tap water once the 30 ml dosage has been fully consumed.

### Monitoring of indirect blood pressure

Baseline blood pressure was measured on the first (day 1) and further monitored on the last day of treatment (day 10). Systolic blood pressure was assessed on preheated conscious rat by tail-cuff method using a non-invasive blood pressure controller coupled to a Powerlab system (AD Instuments Pty Ltd, NSW, Australia). Blood samples were drawn from the rats on the 11^th ^day and sera were obtained by centrifugation of the blood at 4°C. Serum samples were stored in aliquots at -20°C, prior to the experiments.

### Two-dimensional electrophoresis

Two-dimensional electrophoresis (2-DE) was performed as previously described [20]. Serum samples, each containing 100 mg of protein (estimated using BCA™ Protein Assay Kit), were mixed with 450 μl rehydration solution containing 8 M urea, 2% w/v CHAPS, 0.5% v/v IPG buffer, 0.002% w/v bromophenol blue and 10% w/v DTT for 30 min at room temperature. The mixtures were then centrifuged at 1000 rpm for 5 min. The samples were subjected to isoelectric focusing in 24 cm rehydrated precast Immobilline Drystrips at pH 4-7 (GE Healthcare Biosciences, Uppsala, Sweden). For the second dimension, focused samples in the strips were subjected to electrophoresis using 11% polyacrylamide gel in presence of SDS.

### Staining of 2-DE gels

Gels were silver-stained using the protocol described by Heukeshoven and Dernick [21]. A modified silver staining approach was used for gels analysed by mass spectrometry [22].

### Image analysis

Silver-stained gels were scanned using the ImageScanner III. Analysis of serum protein spot volume was performed using the ImageMaster Platinum 7.0 software (GE Healthcare Biosciences, Uppsala, Sweden). Percentage of spot volume contribution refers to the spot volume of a protein expressed as a percentage of the total spot volume of all detected proteins. Results obtained from proteins that were expressed in sera of SDR were used as standard references for comparative purposes.

### Sample preparation for mass spectrometry

Samples were prepared as previously described [23]. Protein spots were manually excised from gels and kept in clean microfuge tubes containing small volumes of Milli-Q water to keep them hydrated. The gel plugs were destained using 15 mM potassium ferricyanide in 50 mM sodium thiosulphate and further reduced and alkylated using 10 mM dithiothreitol and 55 mM iodoacetamide in 100 mM ammonium bicarbonate, respectively. Following thorough washings with 50% acetonitrile (ACN) in 100 mM ammonium bicarbonate and 100% ACN, the gel plugs were dehydrated using vacuum centrifugation. The dried plugs were then incubated in 30 μl of 7 ng/μl trypsin in 40 mM ammonium bicarbonate in 10% ACN solution at 37°C overnight. Peptides were finally extracted using 50% and 100% ACN and subsequently dried using a vacuum centrifuge.

### Mass spectrometry

Dried peptides were reconstituted with 0.1% formic acid and desalted using ZipTip C18 (Millipore, Billerica, USA) according to the protocol described by the manufacturer. The final elution volume following ZipTip cleanup was then mixed with an equal volume of matrix consisting of a saturated solution of α-cyano-4-hydroxycinnamic acid (Sigma Chemical Co., St. Louis, USA) prepared in 50% ACN/0.1% TFA. Each sample was immediately spotted (0.7 μl) onto a stainless-steel sample target plate. The samples were analyzed using the 4800 Plus MALDI TOF/TOF analyzer (Applied Biosystem/MDS Sciex, Toronto, Canada), with the mass standard kit serving as the calibrator for the resulting MS and MS/MS mass spectra scales.

### Protein identification

Data obtained from the MS/MS analysis was generated using peaklist software 4000 Series Explorer (release version 3). The data was exported for search using the MASCOT search engine (Matrix Science, London, UK; release version 2.2) against *Rodentia *entries in the NCBI non-redundant database. Database parameters used were: enzyme/trypsin; one missed cleavage allowed; fixed modification/carbamidomethyl (cysteine); variable modification/oxidation (methionine); mass tolerance for precursor ion/peptide tolerance: 50 ppm and mass tolerance for fragment ion/MS/MS tolerance: 0.1 Da. The cut-off score for accepting individual MS/MS spectra was set at .29 for homology and .38 for matched identity.

### Statistical analysis

ANOVA was used to analyze the significance of differences between SDR, captopril-treated and non-treated SHR. All values are presented as mean ± S.E.M (standard error of the mean). The false discovery rate control was performed using the method of Benjamini and Hochberg [8]. After correction, *p *values of less than 0.00233 were considered significant.

## Competing interests

The authors declare that they have no competing interests.

## Authors' contributions

NA planned the study and drafted the manuscript; NAHA and HM carried out the experiments and analyzed the data; SAK provided advice on mass spectrometry analysis; OHH contributed to the design of the study and critically revised the manuscript; RH and SZH assisted in the measurement of rat blood pressure. All authors read and approved the final manuscript.
